# The Mortality After Release from Incarceration Consortium (MARIC): Protocol for a multi-national, individual participant data meta-analysis.

**DOI:** 10.23889/ijpds.v5i1.1145

**Published:** 2020-01-25

**Authors:** R Borschmann, H Tibble, MJ Spittal, D Preen, J Pirkis, S Larney, DL Rosen, JT Young, AD Love, FL Altice, IA Binswanger, A Bukten, T Butler, Z Chang, C-Y Chen, T Clausen, PB Christensen, GJ Culbert, L Degenhardt, AJE Dirkzwager, K Dolan, S Fazel, C Fischbacher, M Giles, L Graham, D Harding, Y-F Huang, F Huber, A Karaminia, C Keen, FG Kouyoumdjian, S Lim, L Møller, A Moniruzzaman, J Morenoff, E O’Moore, LN Pizzicato, D Pratt, SK Proescholdbell, SI Ranapurwala, ME Shanahan, J Shaw, A Slaunwhite, JM Somers, AC Spaulding, MF Stern, KM Viner, N Wang, M Willoughby, B Zhao, SA Kinner

**Affiliations:** 1 Justice Health Unit, Centre for Health Equity, The University of Melbourne, 207 Bouverie street, Carlton 3010, Melbourne, Victoria, AUSTRALIA; 2 Usher Institute of Population Health Sciences and Informatics, Centre for Medical Informatics, University of Edinburgh, Edinburgh, UK; 3 University of Melbourne, Melbourne School of Population and Global Health, Melbourne, AUSTRALIA; 4 The University of Western Australia, School of Population and Global Health, Nedlands, AUSTRALIA; 5 National Drug and Alcohol Research Centre, UNSW Sydney, Sydney, AUSTRALIA; 6 University of North Carolina at Chapel Hill, North Carolina, USA; 7 University of Melbourne, Melbourne School of Population Health, Melbourne, AUSTRALIA; 8 Yale University School of Medicine and Public Health, New Haven, Connecticut, USA; 9 Kaiser Permanente Colorado, Colorado Permanente Medical Group, USA; 10 Norwegian Centre for Addiction Research, Institute of Clinical Medicine, University of Oslo, Norway; 11 University of New South Wales, Kirby Institute, Sydney, AUSTRALIA; 12 Department of Medical Epidemiology and Biostatistics, Karolinska Institutet, Stockholm, SWEDEN; 13 National Yang-Ming University, Institute of Public Health, TAIWAN; 14 Department of Infectious Diseases, Odense University Hospital and Department of Clinical Research, Faculty of Health Sciences, University of Southern Denmark, DENMARK; 15 Department of Health Systems Science, University of Illinois at Chicago, Chicago, USA; 16 Netherlands Institute for the Study of Crime and Law Enforcement (NSCR), Amsterdam, NETHERLANDS; 17 University of Oxford, Department of Psychiatry, Medical Sciences Division, Oxford, ENGLAND; 18 NHS National Services, Information Services Division, Edinburgh, SCOTLAND; 19 19 Edith Cowan University, School of Arts and Humanities, Joondalup, AUSTRALIA; 20 University of California Berkeley, USA; 21 Taiwan Centers for Disease Control, Taipei, TAIWAN; 22 Cayenne General Hospital, COREVIH Guyane, and Reseau Kikiwi, Cayenne, French Guiana, FRANCE; 23 University of New South Wales, Sydney, AUSTRALIA; 24 McMaster University, Department of Family Medicine, Hamilton, Ontario, CANADA; 25 New York City Department of Health and Mental Hygiene, Bureau of Epidemiology Services, Division of Epidemiology, New York, USA; 26 World Health Organization, Division of Noncommunicable Diseases and Promoting Health through the Life-course, Marmorvej, DENMARK; 27 Somers Research Group, Simon Fraser University, Burnaby, British Columbia, CANADA; 28 University of Michigan, Department of Sociology, USA; 29 Public Health England, London, ENGLAND; 30 Philadelphia Department of Public Health, Philadelphia, PA, USA; 31 University of Manchester, Division of Psychology and Mental Health, School of Health Sciences, Manchester, ENGLAND; 32 North Carolina Department of Health and Human Services, North Carolina, USA; 33 Department of Epidemiology, University of North Carolina at Chapel Hill, USA; 34 Department of Maternal and Child Health, University of North Carolina at Chapel Hill, USA; 35 Centre for Mental Health and Safety, Division of Psychology and Mental Health, University of Manchester, Manchester, ENGLAND; 36 BC Centre for Disease Control, Provincial Health Services Authority, Vancouver, British Columbia, CANADA; 37 Department of Epidemiology, Rollins School of Public Health, Emory University, Atlanta, Georgia, USA; 38 Department of Health Services, University of Washington, Seattle, Washington, USA; 39 Institute of Public Health, National Yang-Ming University, TAIWAN; 40 Murdoch Children’s Research Institute, Centre for Adolescent Health, Melbourne, Victoria, AUSTRALIA

## Abstract

**Introduction:**

More than 30 million adults are released from incarceration globally each year. Many experience complex physical and mental health problems, and are at markedly increased risk of preventable mortality. Despite this, evidence regarding the global epidemiology of mortality following release from incarceration is insufficient to inform the development of targeted, evidence-based responses. Many previous studies have suffered from inadequate power and poor precision, and even large studies have limited capacity to disaggregate data by specific causes of death, sub-populations or time since release to answer questions of clinical and public health relevance.

**Objectives:**

To comprehensively document the incidence, timing, causes and risk factors for mortality in adults released from prison.

**Methods:**

We created the Mortality After Release from Incarceration Consortium (MARIC), a multi-disciplinary collaboration representing 29 cohorts of adults who have experienced incarceration from 11 countries. Findings across cohorts will be analysed using a two-step, individual participant data meta-analysis methodology.

**Results:**

The combined sample includes 1,337,993 individuals (89% male), with 75,795 deaths recorded over 9,191,393 person-years of follow-up.

**Conclusions:**

The consortium represents an important advancement in the field, bringing international attention to this problem. It will provide internationally relevant evidence to guide policymakers and clinicians in reducing preventable deaths in this marginalized population.

**Key words:**

Mortality; incarceration; prison; release; individual participant data meta-analysis; consortium; cohort.

## Introduction

Each year more than 30 million people are released from incarceration globally [1] and this figure is increasing at a rate in excess of population growth [2,3]. The United States (US) imprisons more people than any other country, accounting for more than one-fifth of the estimated 11 million adults incarcerated worldwide on any given day [3]. Due to the large incarcerated population and the rapid turnover of people detained in jails [4], more than 12 million adults cycle through US correctional facilities annually – more than in any other country [5-7]. For many, release from incarceration compounds pre-existing disadvantage as they experience challenges securing stable accommodation and employment, accessing health services, and reconnecting with families, social groups and communities [8]. Further, the risk of premature mortality for adults released from incarceration is substantially higher than in the general population [9]. There are compelling, evidence-based arguments for improving health outcomes and reducing mortality in this population based on human rights, public health, criminal justice, and economic grounds [10,11].

There is an established literature documenting an elevated risk of premature death in the first four weeks after release from incarceration [12-16]. However, the specificity of this increased risk window - and beyond - has not been well characterized. Several barriers have hampered research intended to inform efforts to reduce mortality in this population. First, the absolute number of recorded deaths in most cohorts is relatively small, and some causes of death – such as Human Immunodeficiency Virus (HIV) – remain comparatively rarer in younger populations [17]. As a result, even studies with large samples may lack sufficient statistical power to disaggregate by specific sub-populations (e.g., younger women, men from ethnic minorities), causes of death (e.g., deaths due to suicide, drug overdose, violence, HIV), or windows of time (e.g., the first four weeks after release from incarceration). Yet it is precisely this granularity that is required to meaningfully inform targeted prevention efforts. Second, synthesising findings across published studies is difficult due to the statistical and conceptual heterogeneity observed in published findings to date [9]. Third, researchers may not publish negative findings, or those that were based on insufficient statistical power, and this possible publication bias would threaten the internal validity of findings based exclusively on aggregating published data only. Fourth, jurisdictional data-sharing restrictions have prevented the generation of informative, international benchmarks for mortality after incarceration, such as those produced by the Global Burden of Disease study [18] for population-level morbidity and mortality. Critical questions about the epidemiology of mortality in this vulnerable population therefore remain unanswered.

Few attempts to date have been made to synthesise the literature examining mortality after release from incarceration. Kinner et al. [9] conducted a systematic review of studies examining mortality after release from incarceration, with their review including 29 publications from 25 individual studies, mainly from high-income, Western countries. They documented widely varying mortality estimates, noting that common, avoidable, and significant methodological limitations - and reducible heterogeneity - contributed heavily to this variation. Kinner et al. [9] also identified important knowledge gaps and recommended a targeted meta-analysis of the literature to examine the extent to which the risk of death from specific causes is elevated after release from incarceration, and whether this risk is concentrated in the period immediately following release. Jones et al. [19] conducted a meta-analysis of suicide deaths following release from prison and their findings demonstrated that the risk of suicide in adults following release was 6.8 times that of their non-incarcerated peers (95% confidence intervals: 6.1–7.5). However, their analyses were based on suicide deaths only and were restricted to the five studies (out of nine) with sufficient published data to be included in the meta-analysis. Merrall et al. [12] conducted a meta-analysis of studies examining drug-related deaths following prison release and reported an acute elevation in drug-related deaths in the first two weeks post-release. However, their analyses (a) were restricted to a single cause of death; (b) included only six studies that published their findings in a way that permitted meta-analysis; (c) were restricted to two-week intervals up to 12 weeks post-release, due to power limitations; and (d) did not disaggregate findings by specific sub-populations, limiting the generalisability of the findings. Zlodre et al. [20] conducted a systematic review and meta-analysis of all-cause mortality following release from prison, reporting standardized mortality ratios of 1.0 - 9.4 for males and 2.6 - 41.3 for females, compared to their non-incarcerated peers. The authors included 16 studies from three countries in their analysis, but noted that important gaps remained, such as disaggregation of mortality rates by age, ethnicity, and time since release.

To address the knowledge gaps identified above, we have created the international Mortality After Release from Incarceration Consortium (MARIC). This aim of this paper is to describe the composition of the Consortium, its data, objectives and research methodology. 

### Consortium description

The Consortium is an international, multi-disciplinary and multi-organisational collaboration of researchers, clinicians and policymakers and represents the largest coordinated effort to date worldwide to examine mortality in adults who have experienced incarceration. The Consortium is led by the University of Melbourne in Australia and is funded by Australia’s National Health and Medical Research Council (NHMRC; grant #1120004). It is an open Consortium and welcomes new collaboration proposals from academics, policymakers, clinicians, and service providers worldwide. The Consortium’s current dataset is comprised of 29 cohorts of adults who have experienced incarceration from 11 countries: Australia, Canada, French Guiana (France), Indonesia, Malaysia, the Netherlands, Norway, Scotland, Sweden, Taiwan, and the USA (see Supplementary Figure 1). It combines data regarding individuals who have served (or are serving) a custodial sentence and data regarding individuals who are have been (or are) incarcerated whilst awaiting trial or sentencing. Importantly, the Consortium has access to both published and unpublished data from these cohorts (see Table 1 for descriptive information about each study). Seventeen studies are retrospective cohort studies and twelve are prospective cohort studies (see Appendix 1 for further information about each study’s sampling frame, follow-up time and objectives).

**Figure 1: Sampling and follow-up time by cohort for the 29 cohorts in the Mortality After Release from Incarceration Consortium (MARIC).**
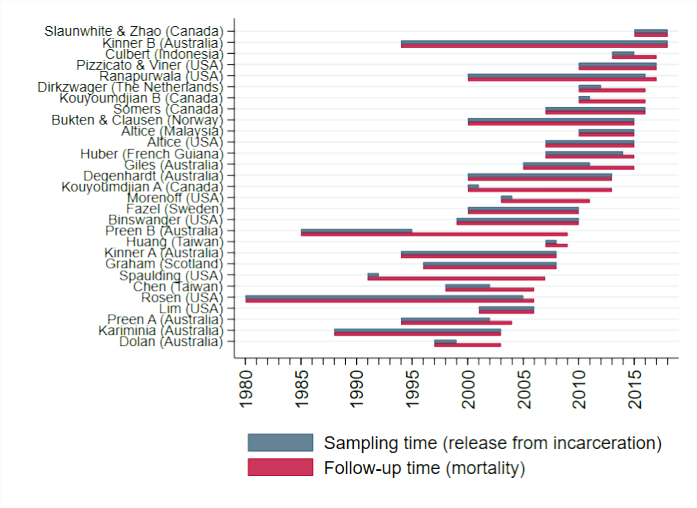


All data have been collected between 1980 and 2017 (see Figure 1) and all studies received ethics approval from relevant local authorities and committees. The total combined sample size of the Consortium is 1,337,993 formerly incarcerated adults, including 153,062 (11%) women. A total of 75,795 deaths have been recorded over 9,191,393 089 person-years of follow-up, and future data linkage updates planned by several cohorts will further expand these totals in the future.

Raw data across the individual cohorts are highly comparable, with many similar measures recorded. Table 2 displays the key variables recorded by each of the 29 cohorts in the Consortium, and how they were measured. All cohort datasets contain data relating to sex, age, date of release from custody, and date and cause(s) of death. Most cohort datasets (25/29; 86%) contain a measure of ethnicity or race. All cohorts use national (n=17), state-based (n=9), or municipal (n=4) death indices (or a combination of these) to ascertain cohort mortality. Causes of death are categorised using the International Classification of Diseases (ICD) [38] codes in all cohorts. Twenty cohorts (69%) contain information regarding dates of all incarceration (and re-incarceration) episodes. Additionally, 16 cohorts (55%) contain information regarding previous substance use problems or treatment (including during incarceration), and nine cohorts contain information regarding previous mental health problems or treatment (including during incarceration).

Individual cohort predictor and outcome data have been collected over a period of 37 years, permitting the detection of changes in mortality trends and determinants over calendar time following release from prison. The data obtained create a large combined sample of individuals with a proportionally large number of person-years of follow-up time, both of which are orders of magnitude greater than that available from any previous study. Accordingly, the Consortium has sufficient statistical power to a) examine specific (including rare) causes of death, and b) conduct meta-regression analyses to consider findings according to key demographic, policy-based and country-level variables, elucidating country-specific structural factors contributing to the observed heterogeneity in mortality estimates. Finally, due to the multi-disciplinary nature of the Consortium, interpretation of findings will also benefit from expert knowledge and experience across a wide spectrum of health and criminal justice settings.

## Outcomes

The main outcome of the Consortium is mortality after release from incarceration. The aims of the Consortium are to: 1) comprehensively establish the incidence and timing of all-cause and cause-specific mortality in adults following release from incarceration internationally; 2) identify risk factors for all-cause and cause-specific mortality following release from incarceration; and 3) examine how risk differs across settings, time, and specific sub-populations. The specific causes of mortality we will examine are:

1. Non-communicable diseases (e.g., asthma [ICD-10: J45-J46], chronic obstructive pulmonary disease [ICD-10: J40-J44; J47], diabetes [ICD-10: E10-E14], cardiovascular disease [ICD-10: I60-I69], cancer [ICD-10 Chapter II]);
2. Alcohol and other drug-related (e.g., opioid overdoses [ICD-10: T40.0-T40.6], alcohol-related deaths [ICD-10: F10], prescribed medications [T43]);
3. Suicide (e.g., self-inflicted injuries [X60-X84]);
4. Infectious diseases (e.g., HIV [ICD-10: B20-B24], hepatitis C [ICD-10: B17], tuberculosis [ICD-10: A15-A19]); and
5. Injuries other than self-inflicted injuries and poisoning (e.g., firearm homicide [ICD-10: X93-X95], assault by bodily force [ICD-10: Y04], legal intervention [ICD-10: Y35-Y36], road traffic accidents [ICD-10: V01]).

## Data Analysis

**Figure 2: Two-step, individual participant data meta-analysis (IPDM-A) methodology.**
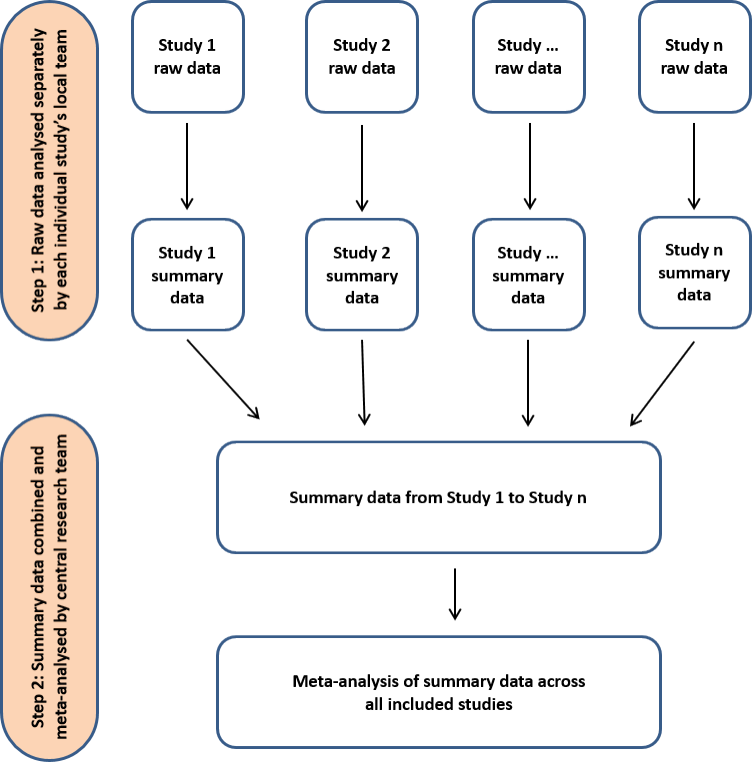


Data analysis for the Consortium is structured around the aims outlined above and will involve a series of two-step, individual participant data meta-analyses (IPDM-A) [39](see Figure 2). In the first stage of the analysis, individual-level data from each cohort’s dataset will be analysed locally by approved analysts in each cohort team according to a pre-specified statistical analysis plan. This plan will define the inclusion and exclusion criteria, specify how data will be harmonised prior to analysis, specify all variables included in the analysis and - for categorical variables - the omitted variables for dummy coding, the specific analytic method (e.g., survival analysis), and how the effect sizes will be presented (e.g., as hazard ratios with standard errors). In the second stage, the results of these analyses will be transferred to a single location (the University of Melbourne) for pooling using random-effects meta-analysis. Again, this will be pre-specified so that only the exposures of interest are examined (with all other predictors considered as confounding factors). All releases from incarceration will be included in future analysis plans, such that one individual can contribute data from multiple incarcerations and releases. To account for multiple releases from incarceration, we will use person-time in the community (follow-up time minus the duration of any periods of re-incarceration) as the denominator when calculating crude and multivariate incidence rates. There are methodological, statistical and clinical advantages to using two-step IPDM-A compared to the traditional meta-analytic approach [39] (see Supplementary Box 1), and this approach has been successfully applied to studies of coronary heart disease [40], vascular mortality [41], and HIV treatment [42] – but not to mortality in adults released from incarceration. With MARIC data stored and governed by data custodians across 11 countries, and ethical and cross-jurisdictional data-sharing restrictions preventing the direct sharing of individual-level data [43], the two-step IPDM-A methodology overcomes these barriers and permits data analysis across all data sources in the Consortium. To account for participant overlap between cohorts – for example, there is a small degree of overlap between the Rosen [34] and Ranapurwala [44] cohorts from the US, and also some overlap between the Dolan [24], Degenhardt [22] and Karaminia [29] cohorts from Australia – we will ensure that all participants, person-time and deaths are only included once prior to conducting any data analysis.

## Strengths and Weaknesses

Strengths of our consortium include its large sample size, its multi-national composition, and its use of ICD codes to assign causes of death. Our Consortium has some limitations. First, 25 of the 29 cohorts (86%) come from high-income countries, with a geographical distribution concentrated in North America (n=12), Australia (n=8), and Western Europe (n=4). While this also represents an advantage, due to the broad similarities of the criminal justice systems in these regions, further data on the health of people who experience incarceration in low- and middle-income countries are urgently needed [45]. To this end, researchers from such under-researched settings are encouraged to join the Consortium and a) contribute extant data, or b) develop new datasets to address this limitation. Second, consistent with international incarceration rates [46], men make up a large proportion (88%) of the combined MARIC sample. However, the Consortium provides an unprecedented opportunity to examine mortality in a large sample of women (N=153,062) released from incarceration across eight countries. Third, the Consortium includes cohorts with a degree of heterogeneity and this is likely to have impacted the observed mortality estimates. For example, participants in several cohorts all have as inclusion criteria a history of opioid dependence [22, 24, 47] and/or a diagnosis of HIV [28, 47, 48], contributing to an increased risk of mortality. The impact of this cohort heterogeneity will be explored by conducting sensitivity analyses which exclude selected cohorts.

### Conclusion and future research

The disproportionate rates of premature mortality experienced by adults released from incarceration [9] represents an unnecessary and preventable loss of life. The MARIC study is the largest coordinated effort worldwide to rigorously and comprehensively examine mortality in a population who often experience profound disadvantage, complex physical and mental health problems, in addition to an increased risk of preventable mortality.

Importantly, incarceration itself is a high-risk event for morbidity and mortality outcomes [49, 50] and international efforts focussing on diverting vulnerable people away from incarceration are warranted to reduce the high rates of incarceration currently observed worldwide [3]. Simultaneously, incarcerated people at increased risk of mortality following release must be identified prior to release so that appropriate evidence-based interventions can be implemented in a timely manner. Identifying people who are disproportionately likely to experience challenges relating to accommodation, food security, employment, and substance use following release from incarceration will also likely identify those at an increased risk of premature mortality. Another possible avenue is the development and implementation of clinical prediction rules to identify those at highest risk of mortality following release from incarceration, comparable to Fazel and colleagues’ prediction rule to identify people at highest risk of violent recidivism after prison release [51]. Similar rules for mortality, if they could be developed, would potentially be inexpensive and scalable, and findings could be used to link people identified as being at increased risk with appropriate treatment and care during the incarceration period, and ongoing care after release.

The MARIC Consortium represents an opportunity to substantially improve the evidence base regarding mortality in adults released from incarceration and produce targeted, globally relevant evidence on the epidemiology of mortality in this population. The overarching aim of the Consortium is to substantially increase the accuracy, precision, clinical relevance and translational impact of research on mortality in adults following release from incarceration across countries. Findings and recommendations from the Consortium will lay the foundation for policy reform, targeted clinical intervention, and rigorous evaluation of scalable interventions that have the potential to reduce the unnecessary wastage of lives after release from incarceration internationally. 

## How can I find out more or get involved?

Further information about the MARIC Consortium is located at: https://mspgh.unimelb.edu.au/research-groups/centre-for-health-equity/justice-health-unit/mortality-after-release-from-incarceration-consortium-maric-study. Specific inquiries, including collaboration proposals, can be directed to the Consortium’s Chief Investigator, Dr. Rohan Borschmann (rohan.borschmann@unimelb.edu.au). The Mortality After Release from Incarceration Consortium includes all authors listed above, in addition to Trudi Cooper, Neil Drew, Lisa Duffy, Michael Farrell, Cath Ferguson, Natalie Gately, Natasa Gisev, Ann-Claire Larsen, Jo Kimber, Richard Mattick, Paul Nieuwbeerta, Moira Sim, Di Twigg, and Jacqui Whale.

## Funding and acknowledgements

The MARIC study is funded by Australia’s National Health and Medical Research Council (NHMRC, project #1120004). The authors wish to acknowledge the assistance of the Western Australian Department of Justice in the conduct of the research, along with all organisations, government departments, and other entities worldwide that have contributed to this research. The material published herein cannot be considered as either endorsed by the Western Australian Department of Justice or an expression of the policies or view of the Department. The opinions generated in this study do not represent the British Columbia Ministry of Health or any data stewards. Any errors of omission or commission are the responsibility of the research team.

## Authors’ contributions:

RB, SK, MS, JP, SL, DP, DR, EO, and LM obtained funding for the Consortium. RB produced the first draft of the manuscript. HT, JY and CK produced the tables and figures. All authors contributed to subsequent iterations of the manuscript and approved the final manuscript prior to submission.

## Patient and Public Involvement

We did not involve patients or the public in our work.

## Ethics Statement

Ethics approval was granted for all 29 individual cohorts in the consortium, and no further approval was required for the broader collaborative study.

## Supplementary Material

Tables

Supplementary File
