# Supplementary material for: The Mortality After Release from Incarceration Consortium (MARIC): Protocol for a multi-national, individual participant data meta-analysis
Source: Int J Popul Data Sci. 2020 Jan 25;5(1):1145. doi: 10.23889/ijpds.v5i1.1145 (PMC7473255; doi:10.23889/ijpds.v5i1.1145)
Supplement: Tables [file ijpds-05-1145-s001.pdf]

Table 1. Description of cohorts included in the Mortality After Release from Incarceration Consortium (MARIC).

Chief investigator(s)	Country	Sample size	Number of males; N (%)	Deaths (% of cohort)	Start year	End year	Median follow-up time in years (25% & 75% IQR*; minimum & maximum values)	Follow-up time (person-years)	Crude mortality rate per 1,000 person-years (95%CI)	Death register used
Altice	Malaysia	291	291 (100)	62 (21)	2010	2014	3.1 (IQR: 2.1 - 4.0; range: 0.1 - 6.4)	871	71 (54-91)	National
Altice	USA	1,350	1043 (77)	184 (14)	2007	2014	5.2 (IQR: 3.0 - 6.7; range: 0 - 7.9)	6,480	28 (24-33)	National
Binswanger	USA	76,208	63,979 (84)	2,462 (3)	1999	2009	4.1 (IQR: 2.1 - 6.4; range: 0.0 - 10.5)	334,238	7 (7-8)	National
Bukten & Clausen [13]	Norway	92,554	83,228 (90)	6,313 (7)	2000	2014	3.6 (IQR 1.4 - 7.9; range: 0 - 15.0)	751,259	8 (8-9)	National
Chen [21]	Taiwan	26,668	17,780 (83)	533 (2)	1998	2005	5.7 (IQR: 4.7 - 6.6; range 0 - 7.6	78,527	7 (6-7)	National
Culbert	Indonesia	68	68 (100)	5 (7)	2013	2016	0.1 (IQR: 0.0 - 0.7; range: 0.0 – 1.2)	21	238 (77-556)	Municipal
Degenhardt [22]	Australia	16,453	12,945 (79)	1,050 (6)	2000	2014	6.3 (IQR: 3.5 - 8.2; range: 0 - 9.3)	100,978	10 (10-11)	National
Dirkzwager & Nieuwbeerta [23]	Netherlands	1904	1904 (100)	42 (2)	2010	2015	4.2 (IQR: 3.9 - 4.6; range: 0.2 - 5.0)	7,626	6 (4-7)	National
Dolan [24]	Australia	369	369 (100)	25 (7)	1997	2002	0.3 (IQR: 0.1 - 0.8; range: 0 - 9.3)	2,088	12 (8-18)	National
Fazel [25]	Sweden	47,326	42,840 (93)	2,874 (6)	2000	2009	5.1 (IQR: 2.6 - 7.5; range: 0 - 10.0)	238,457	12 (12-13)	National
Giles	Australia	14,643	12,897 (88)	752 (5)	2005	2014	2.3 (IQR: 0.4 - 3.8; range: 0 - 11.2)	33,332	23 (21–24)	National
Graham [26]	Scotland	76,627	68,315 (89)	4,414 (6)	1996	2007	6.8 (IQRs: 3.4 - 10.0; range: 0 - 12.0)	506,000	9 (8–9)	National
Huang [27]	Taiwan	4,357	3,851 (88)	142 (3)	2007	2008	1.5 (IQR: 1.5 - 1.5; range: 0 - 1.5)	6,253	23 (19–27)	National
Huber [28]	French Guiana (France)	147	120 (82)	12 (8)	2007	2014	3.9 (IQR: 1.7 - 5.5; range: 0 - 7.8)	284	42 (22–74)	Municipal

Kariminia [29]	Australia	82,650	73,984 (90)	8,666 (6)	1988	2002	7.4 (IQRs: 3.6 - 11.2; range: 0 - 15.0)	619,845	8 (8–9)	National
Kinner ‘A’ [30]	Australia	42015	37,039 (88)	2,203 (5)	1994	2007	7.6 (IQR: 3.5 - 10.3; range: 0 - 14.0)	297,116	7 (7–8)	National
Kinner ‘B’	Australia	13,188	11,650 (88)	481 (4)	1994	2017	7.9 (IQR: 3.4 - 13.5; range: 0 - 23.0)	114930	4 (4–5)	National
Kouyoumdjian ‘A’	Canada	48,166	43,419 (90)	4,126 (9)	2000	2012	12.6 (IQR: 10.3 - 12.6; range: 0.0 - 13.0)	500,942	8 (8–9)	Provincial
Kouyoumdjian ‘B’	Canada	48,861	42,754 (88)	1,869 (4)	2010	2015	5.6 (IQR: 5.3 - 5.8; range: 0.0 - 6)	265,919	7 (6–7)	Provincial
Lim [15]	USA	155,272	137,161 (88)	473 (<1)	2001	2005	2.5 (IQR: 1.1 - 3.8; range: 0 – 5.0)	379,363	1 (1-1)	Municipal
Morenoff	USA	11,064	10,202 (92)	533 (5)	2003	2010	6.5 (IQR: 6.1 - 6.8; range: 0 - 7.1)	55,296	10 (9–10)	National
Pizzicato & Viner	USA	82,780	66,409 (80%)	2,522 (3)	2010	2016	4.16 (IQR: 3.2 - 2.4; range: 0 - 7.0)	325,393	8 (7–8)	Municipal, State
Preen ‘A’ [31]	Australia	16,162	14,232 (88)	698 (4)	1994	2003	6.4 (IQR: 3.9 - 9.2; range: 0 - 10.3)	102,472	7 (6–7)	State
Preen ‘B’ [32]	Australia	6,041	5424 (90)	645 (11)	1985	2008	18.2 (IQR: 14.9 - 21.3; range: 0 - 23.9)	106,430	6 (6–7)	State
Ranapurwala [33]	USA	229,274	197,656 (86)	14,086 (6)	2000	2016	8.5; (IQR: 5.0 - 12.3; range: 0 - 17.0)	1,975,274	7 (7–7)	State
Rosen [34]	USA	168,00179	168,001 (100)	15,673 (8)	1980	2005	10.3 (IQR: 4.7 - 16.0; range: 0-26-)	1,822,869	9 (8–9)	State
Slaunwhite & Zhao [35]	Canada	9,993	8684 (87)	351 (4)	2015	2017	4.3 (IQR: 2.0 – 6.7; range: 0 – 8.0)	42,665	8 (8-8)	Provincial
Somers [36]	Canada	42,625	37,176 (80)	1,430 (3)	2001	2015	4.8 (IQR: 2.6 - 6.6; range: 0 – 8.2)	195,171	7 (7–8)	Provincial
Spaulding [37]	USA	20,743	19,511 (94)	3,169 (15)	1991	2006	14.5 (IQR: 8.7 – 17.9; range: 0 – 19.5)	321,294	10 (9–10)	National
**TOTAL**	- - -	**1,337,993**	1,185,331 (89)	**75,795 (6)**	1980	2017	- - -	**9,191,393**	**8 (8–8)**	- - -

* IQR = interquartile range

Table 2. Variables recorded by individual cohorts in the Mortality After Release from Incarceration Consortium (MARIC).

Chief investigator(s)	Unselected study population of adults released from prison	Gender	Race/ethnicity categories	Age range (years)	Cause of death			Re-incarceration data	Data linkage method	Mental Illness measurement	Substance use measurement
					Underlying cause	*Contributing cause(s)*	*ICD 9/10*				
Altice (Malaysia)	Males with HIV and opioid dependence	Males only			√					Self-report	Self-report
Altice (USA)	Adults with HIV	√	White; Black; Hispanic; Other		√	√	10	√	P	Linked data	Linked data
Binswanger	√	√	White, Non-Hispanic; African American, Non-Hispanic; Hispanic; Asian, non-Hispanic; Other	18-85	√	√	10	√	P	Linked data	Linked data
Bukten & Clausen	√	√	Country of Birth		√	√	10	√	D		
Chen	Adults incarcerated for drug-related offences	√		18-75	√		9	√	D		Linked data
Culbert	Males with HIV	Males only	Austronesian (Javanese, Sundanese, Betawi)	21-45	√						Self-report
Degenhardt	Adults with opioid dependence treated with OST	√	Indigenous; Non-Indigenous	14-64	√	√	10	√	P		Self-report
Dirkzwager & Nieuwbeerta	√	Males only	Ethnic or Migration Background (Y/N)	18-65	√	√	10	√	D	Self-report	Self-report
Dolan	Treatment-seeking males who use heroin	Males only	Indigenous; Non-Indigenous	18-46	√		10	√	P		
Fazel	√	√	Born in Sweden; Born outside Sweden	15-85	√	√	10		D	Linked data	Linked data
Giles	√	√	Indigenous; Non-Indigenous	17-87	√	√	10	√	P		
Graham	√	√	White; Other; Missing	14-90	√	√	9/10	√	P	Linked data	Linked data

Huang	Adults incarcerated for heroin-related offences	√	Asian	19-74	√	√	9	√	D		Linked data
Huber	Adults with HIV incarcerated for >30 days	√*	Born in France; Migrants	23-60	√					Medical records	Medical records
Kariminia	√	√	Aboriginal; Non-Aboriginal; Other English speaking countries; Asian; Other non-English speaking countries; Unknown	17-86	√		9/10	√	P	Linked data	
Kinner 'A'	√	√	Indigenous; Non-Indigenous	16-90	√	√**	9/10	√	P		
Kinner 'B'	Adults with previous youth justice involvement	√	Indigenous; Non-Indigenous	16-40	√	√	9/10	√	P		
Kouyoumdjian 'A'	√	√	Aboriginal; Black; Declined; East Asian, Hispanic; Other; South Asian; Southeast Asian; Unknown; West Asian/Arab; White	16-88	√	√	9	√	D/P	Linked data	
Kouyoumdjian 'B'	√	√	Aboriginal; Black; Declined; East Asian, Hispanic; Other; South Asian; Southeast Asian; Unknown; West Asian/Arab; White	17-98	√	√	9	√	D/P		
Lim	√	√	Non-Hispanic White; Non-Hispanic Black; Hispanic; Asian; Other	16-89	√		10	√	P		
Morenoff	Adults paroled from state prisons	√			√			√	P		
Pizzicato & Viner	√	√	Non-Hispanic White; Non-Hispanic Black;	15-84	√	√	10	√	P	Linked data	

			Hispanic; Other/Unknown								
Preen 'A'	√	√	Indigenous; Non-Indigenous	16-87	√	√	9/10	√	P	Linked data	Linked data
Preen 'B'	√	√	Indigenous; Non-Indigenous	18-87	√	√	9/10	√	P	Linked data	Linked data
Ranapurwala	√	√	Non-Hispanic White; Non-Hispanic Black; Hispanic; Asian; Other	18-92	√	√	10	√	P	Linked data	Linked data
Rosen	√	Males only	Black; White; excluded those not classified as black or white	20-69	√	√	9/10		P		
Slaunwhite & Zhao	√	√		18-91	√	√	10	√	P	Linked data	Linked data
Somers	√	√	Caucasian; Aboriginal; Asian; Black; Hispanic	18-91	√	√	9/10	√	**P**	Linked data	Linked data
Spaulding	√	√	Black; Non-Black	13-91	√	√	9/10	√	P	Linked data	

* All recorded deaths were male
** Contributing causes available from 1997
P = Probabilistic linkage; D = Deterministic linkage
