# Supplementary material for: The Mortality After Release from Incarceration Consortium (MARIC): Protocol for a multi-national, individual participant data meta-analysis
Source: Int J Popul Data Sci. 2020 Jan 25;5(1):1145. doi: 10.23889/ijpds.v5i1.1145 (PMC7473255; doi:10.23889/ijpds.v5i1.1145)
Supplement: Supplementary File [file ijpds-05-1145-s002.pdf]

Supplementary material

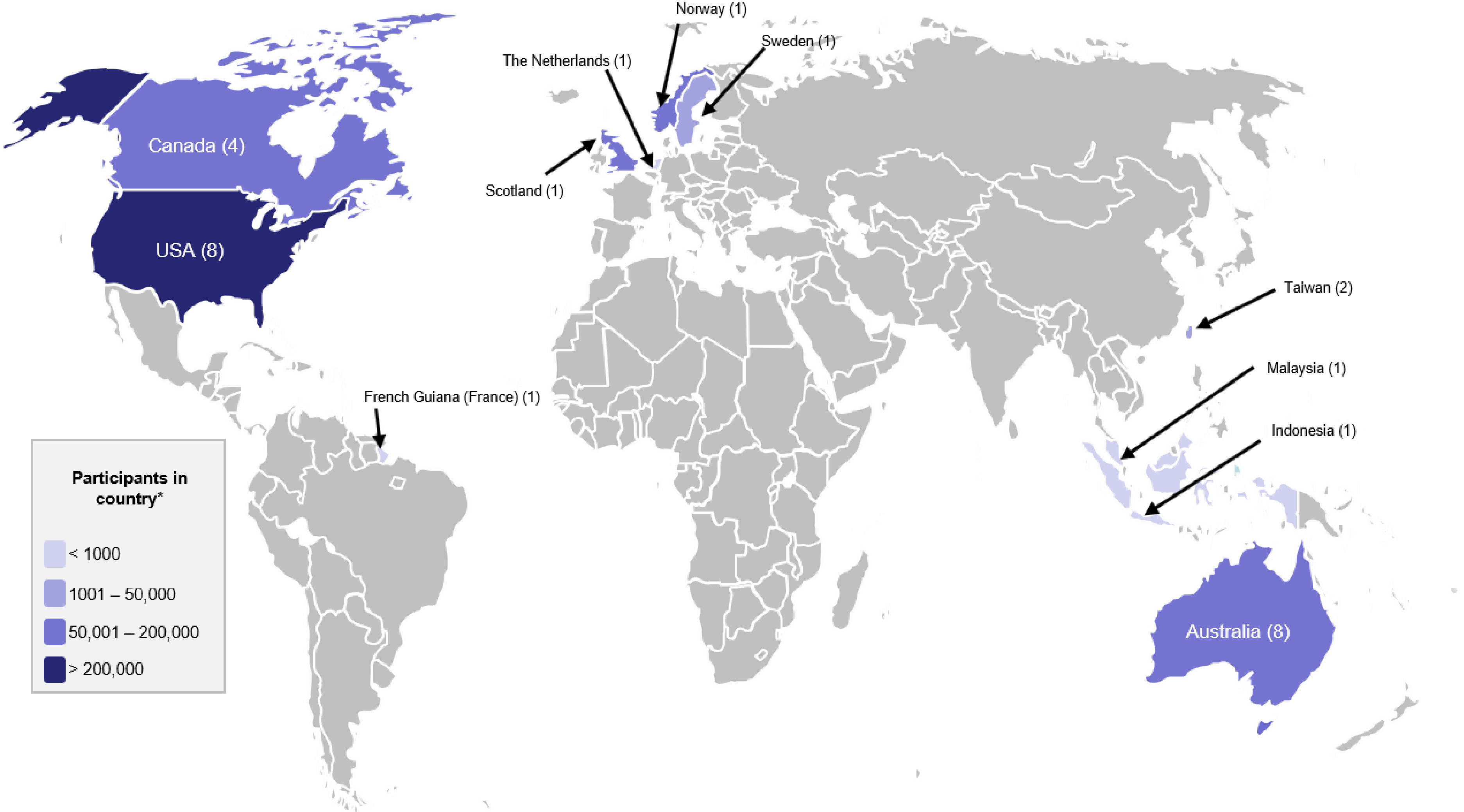

Supplementary Figure 1. Map of countries contributing data to the Mortality After Release from Incarceration Consortium (MARIC) and the number of cohorts and participants from each country.

Supplementary Box 1. Advantages of the two-step, individual participant data meta-analysis (IPDM-A) method (1).

1) Methodologically, it allows for unpublished data to be analysed and incorporated (thereby reducing publication bias), and consistent inclusion/exclusion criteria to be applied across studies;
2) Statistically, it increases power relative to individual studies and is, simultaneously, likely to reduce statistical heterogeneity because many of the sources of heterogeneity can be addressed in the first step through the process of standardization;
3) It allows for more meaningful synthesis of findings because the approach to data analysis is standardized across studies and allows effect estimates to be generated for specific sub-populations, time periods, and causes of death;
4) It permits consistent adjustment for confounding factors that may explain differences in findings across studies, and increases the clinical relevance of findings by providing the opportunity to explore nuanced clinical questions that cannot be answered by the individual studies alone;
5) It permits use of meta-regression analyses to examine the impact of policy-relevant structural- and country-level parameters;
6) It overcomes the privacy hurdles associated with sharing individual level data across jurisdictions, as data are analysed locally in the first step;
7) In contrast to the traditional meta-analytic approach, involving the original study investigators provides them an opportunity to convey the nuances of their data, so that these nuances can be considered when developing the two-step IPDM-A and interpreting the results.

Appendix 1. Descriptions of the individual studies in the Mortality After Release from Incarceration Consortium (MARIC), and peer-reviewed papers relating to mortality published from each study to date.

1. **Altice; USA**

The Connecticut Prison/Jail Post-Release HIV Cohort is a comprehensive state-wide cohort that includes all adults with a confirmed HIV diagnosis (N=1,350) released from a Connecticut prison or jail during an 8-year period (1 January 2007 to 31 December 2014). Data were obtained from the Connecticut Department of Public Health's 'enhanced HIV/AIDS Reporting System' (eHARS), cross-referenced with the United States National Death Index, and then matched to Connecticut Department of Correction records based on name, date of birth, race, gender, and prison/jail number. The Department of Correction databases capture date of entry into a prison or jail and date of release, both for sentences served and time spent on remand, and all medications prescribed during incarceration. The Department of Public Health data capture information regarding initial HIV diagnosis as well as all monitoring HIV ribonucleic acid (RNA) viral levels drawn thereafter until death. For deaths that occurred within Connecticut, primary and secondary causes of mortality were assigned using ICD-10 codes. Most cohort members (77.3%) were male. During a median follow-up of 5.2 years, there were 184 deaths (153 men, 31 women). This cohort has served as the basis for several retrospective studies assessing post-release linkage to HIV care, retention in HIV care, and causes and predictors of all-cause mortality among all people living with HIV formerly incarcerated in Connecticut during 2007-2014. Further information is available by contacting Dr. Frederick Altice (frederick.altice@yale.edu).

2. **Altice; Malaysia (2)**

Project Harapan is a prospective clinical trial conducted among people living with HIV and opioid dependence who transitioned from prison to the community in Malaysia between 2010 and 2014. The trial involved two interventions: 1) a within-prison initiation of methadone maintenance therapy; and 2) an evidence-based behavioural intervention adapted to the Malaysian context (the Holistic Health Recovery Program for Malaysia, HHRP-M). Participants were recruited and received the interventions while incarcerated, and were followed for 12 months after release to assess post-release HIV transmission risk

behaviours and other health-related outcomes. Mortality data were collected by linking participants to a national death registry. Although the length of follow-up time differed between participants, this registry was queried more than a year after the last participant was released from prison. The sample of 291 included in the MARIC cohort excludes five participants who died while incarcerated and five for whom survival data were not available in the death registry. Further information is available by contacting Dr. Frederick Altice (frederick.altice@yale.edu).

3. Binswanger; USA (3-10)

The Washington State Prison Release study, led by Dr. Ingrid A. Binswanger, is a longitudinal cohort of 76,208 adults released from state prisons in Washington, USA, between 1 July 1999 and 31 December 2009. The study links data to the U.S. National Death Index, and identified 2,462 deaths after release from prison over a mean follow-up time of 4.4 years. The study was also the basis for a nested case control study examining detailed medical, substance use treatment, and administrative records to identify risk factors for all-cause and overdose mortality. The Washington State Prison Release study was originally funded by the Robert Wood Johnson Foundation and subsequently by the National Institute on Drug Abuse (R21DA031041). It was conducted with the collaboration of Dr. Marc Stern and the Washington State Department of Corrections. Further information is available by contacting Dr. Ingrid A. Binswanger (ingrid.a.binswanger@kp.org).

4. Bukten & Clausen; Norway (11)

The Norwegian Prison Release study, led by Dr. Anne Bukten and Professor Thomas Clausen, is a national study which includes all persons (N=92,554) released 153,604 times from all Norwegian prisons during a 15-year period (1 January 2000 to 31 December 2014). Data were obtained from the Norwegian prison registry, linked to the Norwegian Cause of Death Registry (General Mortality Registry) (2000-2014) based on unique 11-digit personal identifiers. The prison registry captures date of admission and date of release, both for sentences served and time spent on remand. All-cause and cause-specific mortality were identified according to ICD-10 codes. The majority of the cohort (n= 83,228; 90%) was male. In total, there were 6,313 deaths (5,770 men, 543 women) during a mean follow-up of 4.9

years. The study cohort was the basis for an observational study investigating overdose death in the immediate period following release from prison and up to six months. The Norwegian cohort to be included in the MARIC study may be updated in the future to accommodate longer inclusion times or observation times including updated mortality data. Further information is available by contacting Dr. Anne Bukten (anne.bukten@medisin.uio.no).

5. **Chen; Taiwan (12)**

This study was based on the 1998–2001 judiciary records relating to drug-related offences in Taiwan, and data were provided by the Ministry of Justice's Statistics Office. The study was supported by the Ministry of Justice (P950823) and the National Health Research Institutes (98A1-PHPP44-021). The cohort comprised 26,668 adults charged with drug-related offences who (i) were aged 18 or over; (ii) had served a sentence of at least one day in a correctional facility (i.e., jail, prison, or an observation / rehabilitation institution); (iii) were involved in 'Schedule I or II' drugs; and (iv) were alive at their sentence end date. Death data were provided by the 1998-2005 death registration records, maintained by the Department of Health's Division of Statistics. Causes of death were coded and extracted according to the ICD-9. Males made up more than 80% of the sample, and approximately three quarters were aged 18-29 years and charged with 'Schedule II' drug-related offences. Within the 3-year period following release, 2% of cohort members died and almost half were re-incarcerated at least once. Further information is available by contacting Prof. Chuan-Yu Chen (cychen@nhri.org.tw).

6. **Culbert; Indonesia (13)**

This study, led by Dr. Gabriel Culbert, examined all-cause mortality in HIV-infected Indonesian male adults whilst incarcerated and up to 24 months post-release. A total of 102 randomly selected HIV-infected adults incarcerated in one of two prisons in Jakarta, Indonesia, completed surveys and were followed up for two years (until study completion), or until they died or were lost to follow-up. Death dates were determined from medical records and interviews with immediate family members. During 103 person-years of follow-up, 15 deaths occurred (including ten in prison). The crude mortality rate within prison

(125.2 deaths per 1,000 person-years) was surpassed by the crude mortality rate (215.7 deaths per 1,000 person-years) in cohort members released from prison. The most common cause of death was HIV-associated opportunistic infections. Receipt of addiction treatment was associated with longer survival (HR 0.1, 95% CI 0.01–0.9; *P*=0.03), although treatment with antiretroviral therapy (ART) or methadone was not. Further information is available by contacting Dr. Gabriel Culbert (gculbert@uic.edu).

7. **Degenhardt; Australia (14-18)**

The Opioid Substitution Therapy (OST) and Crime study, led by Prof. Louisa Degenhardt, was funded by Australia's National Health and Medical Research Council (NHMRC; APP1005668) and the Australian Institute of Criminology. The study included data from all people prescribed opioid substitution therapy for the treatment of opioid dependence in New South Wales (NSW), Australia, between 1985 and 2010 (N=48,069). These data were probabilistically linked to incarceration records (available from 2000 onwards) and the National Death Index up to 31 March 2012. Linkage was based on full name, aliases, sex and date of birth. The cohort for the MARIC study includes 16,453 people with opioid dependence who were released from incarceration at least once between January 2000 and March 2012 (total releases: 60,161). The majority of the cohort is male (78.7%) and 29.9% were identified as Indigenous in at least one dataset. During 100,978 person-years in the community following index release from incarceration, there were 1,050 deaths (crude mortality rate 10.4 per 1000 person-years; 95% confidence interval 9.8, 11.0 per 1000 person-years). Further information is available by contacting Prof. Louisa Degenhardt (l.degenhardt@unsw.edu.au).

8. **Dirkzwager & Nieuwbeerta; Netherlands (19)**

The Prison Project, led by Dr. Anja Dirkzwager and Prof. Paul Nieuwbeerta, is a cohort study on the effects of imprisonment in the Netherlands on the further life-course of the adults who experienced incarceration and their families. A total of 1,904 males (all of whom were aged 18-65, born in the Netherlands, and placed in pre-trial detention between October 2010 and April 2011) were interviewed three weeks after their arrival in detention. These men were interviewed on multiple occasions both during imprisonment and after release. Detailed information (both self-reported and from administrative data) was collected on

numerous life domains (e.g. employment, housing, social networks, health) and on characteristics of their time during incarceration (e.g. aggression by incarcerated peers, visits, misconduct). Cohort members were an average of 30 years of age at the time of admission, 46% were detained for a violent offence and 31% for a property offence. Information on dates and causes of death were obtained through linkage with the Statistics Netherlands Death Index. Four respondents died during imprisonment and at the end of follow-up (May 2015) 37 persons were still incarcerated, leaving 1,863 persons for whom mortality after release from prison could be examined. At the time of writing, 42 deaths have been observed during a mean follow-up of 4.2 years. Further information is available by contacting Dr. Anja Dirkzwager (adirkzwager@nscr.nl).

9. Dolan; Australia (20)

This study, led by Prof. Kate Dolan, examined whether methadone maintenance treatment reduced heroin use, syringe sharing and HIV or hepatitis C incidence among incarcerated adults in the state of New South Wales, Australia. All eligible incarcerated adults seeking drug treatment were randomized to methadone or a waitlist control group from 1997 to 1998 and followed up after 5 months, 4 years and 10 years. Of 593 eligible adults, 382 (64%) were randomized to methadone maintenance treatment (MMT; n=191) or control (n=191). Of these, 369 were released from prison at least once. A total of 129 treated and 124 control participants were followed up at five months. Heroin use was significantly lower among treated than control participants at follow up. Treated participants also reported lower levels of drug injection and syringe sharing at follow-up. There was no difference in HIV or hepatitis C incidence. At 4-year follow-up, there were no deaths recorded while participants were enrolled in MMT. Seventeen participants died while out of MMT, representing an untreated mortality rate of 2.0 per 100 person-years (95% CI, 1.2–3.2). At 10 year follow-up, 28 participants (7.5%) had died (25 of which occurred in the community after release from prison); half of these were classified as accidental drug-related deaths. Participants died at six times the rate of their age- and sex-matched peers in New South Wales during the same period. Mortality was significantly reduced while participants were in treatment or in prison. There were some indications that the four week period post-release was a time of increased risk of mortality, particularly from drug overdose, but the comparison to mortality during all other time at liberty did not reach statistical significance.

At the time of writing, a 20-year follow-up is in preparation. Further information is available by contacting Prof. Kate Dolan (k.dolan@unsw.edu.au).

10. Fazel; Sweden (21)

This study, led by Prof. Seena Fazel, linked six population-based registers in Sweden: (1) the National Crime Register, which includes detailed information about all criminal convictions since 1973; (2) the National Patient Register (NPR), which provides diagnoses for all inpatient psychiatric hospital admissions in Sweden since 1973 and outpatient care since 2001; (3) the Migration Register, which supplies information on dates for migration in or out of Sweden; (4) the Cause of Death Register, which contains information on dates and causes of all deaths since 1958; (5) the Multi-Generation Register, which contains information about biological relationships for all individuals living in Sweden since 1933; and (6) the Longitudinal Integration Database for health insurance and labour market studies, which contains yearly assessments of income, marital status, employment status, and education for all individuals 15 years or older since 1990. In Sweden, all residents (including immigrants) have a unique personal identifier used in all national registers, thus enabling data linkage. A cohort of all adults who had been imprisoned since January 1, 2000 and released before December 31, 2009 (N=47,326) was selected. The cohort was followed from the day of release until new offence conviction, death, emigration or end of the study (December 31, 2009). Cohort members with any psychiatric diagnoses before release from prison were identified, based on International Classification of Diseases (ICD) codes. All-cause and cause-specific mortality (according to ICD chapters) were identified, in addition to criminality (violent offences, and all offences [including violent offences]). Further information is available by contacting Prof. Seena Fazel (seena.fazel@psych.ox.ac.uk).

11. Giles; Australia

This project, led by Associate Professor Margaret Giles of the Centre for Innovative Practice at Edith Cowan University, Western Australia (WA), is a continuation of an ongoing project into the post-release outcomes for incarcerated and formerly incarcerated adults in WA. The overarching project uses linked data from the WA Department of Justice (previously Department of Corrective Services; prison history and prison study data), Centrelink (welfare payments data), and the Australian Institute of Health and Welfare (death data).

The data linkage required that each of the data custodians create the SLK581 statistical linkage key in their databases prior to supplying de-identified datasets to the research team. To date, the project has received funding from Edith Cowan University, the WA Department of Corrective Services and the Australian Institute of Criminology. The linked dataset contains prison history and correctional education data for 14,643 adults who had been imprisoned between 1 July 2005 and 30 June 2010. It also includes 137,026 welfare payment records from March 1975 to May 2013, as well as death records for the 752 adults (5.1% of the 14,643) following release from prison who died between 2005 and 2014. Preliminary analysis of the linked dataset shows that 12% of all deaths occurred within six months of release for adults released from their most recent term; the three most prevalent causes of death were 'intentional self-harm by hanging, strangulation and suffocation' (15% of deaths), 'chronic ischaemic heart disease' (9.6% of deaths) and 'accidental poisoning by, and exposure to, other and unspecified drugs, medicaments and biological substances' (7.6% of deaths). The proportion of deaths was lower for cohort members that studied whilst in prison (4.9%) compared with those that did not (5.7%; Chi square=3.7955; p=0.051). Further information is available by contacting A/Prof. Margaret Giles (m.giles@ecu.edu.au).

12. Graham; Scotland (22, 23)

This study, led by Dr. Lesley Graham, is a data linkage study of mortality among adults released from prison in Scotland. It was funded by the Scottish Chief Scientist Office (CZG/2/282). The cohort included all adults imprisoned in Scotland for the first time during the 12 years between 1 January 1996 and 31 December 2007 (inclusive). Participant identifiers held in Scottish Prison Service databases were linked using standard probabilistic methods to death registration records provided to National Health Service (NHS) National Services Scotland by the National Records of Scotland. The cohort included 76,627 adults, of which 4,414 died by the end of follow up on 31 December 2007. The total follow-up time was 454,800 person-years among 68,315 males and 51,200 person-years among 8,312 females, with a median follow up time of 6.9 years for males and 6.1 years for females. The cohort was predominantly male (89%), with a median age of 26.7 in males and 27.0 in females. Further information is available by contacting Dr. Lesley Graham (lesley.graham@nhs.net).

13. Huang; Taiwan (24)

The Taiwan Amnesty cohort study, led by Professor Kenrad E. Nelson and Dr. Yen-Fang Huang, is a data linkage study of mortality in adults released from prison in Taiwan. It was funded by National Institute on Drug Abuse in the USA and Taiwan Centers for Disease Control. The cohort includes the 4,357 adults who were imprisoned for offences related to illegal heroin use and released from prison on the same day, 16 July 2007 in Taiwan. Mortality between July 2007 and December 2008 was determined by linking the National Death Registry with the Methadone Maintenance Treatment (MMT) database. The sample is predominantly male (88%) and aged <49 at index release (90%; median age 37 years). A total of 142 deaths were recorded. The total follow-up time was 6,253 person-years (median 1.4 years per person), and the total community follow-up time (excluding subsequent periods of re-incarceration) was 5,918 person-years. A total of 1,982 (46%) participants enrolled in MMT, of which 1,282 (65%) discontinued MMT after enrolling. The mortality ration among those who continued attending MMT (MR = 0.24/100 person-years) was lower than those who never enrolled in MMT (MR = 2.6/100 person-years) or those who enrolled but discontinued MMT (MR = 7.0/100 person-years) (RR = 0.07). Further information is available by contacting Dr. Yen-Fang Huang (emily@cdc.gov.tw).

14. Huber; French Guiana (France) (25)

This study examined mortality during the first seven years after release from incarceration for HIV-positive adults from the French Guiana correctional facility. All HIV-infected adults released from an incarceration of 30 days or more between 1 January 2007 and 31 December 2013 were enrolled in a retrospective cohort study. The cohort included 147 adults, 120 (81.6%) of whom were male. The median age at release was 37.3 years. Thirty-eight cohort members (25.8%) were homeless and 103 (70.1%) reported substance abuse issues, including 50 (34.0%) who used crack-cocaine. On admission, 115 (78.1%) had an early HIV-stage infection (Centers for Disease Control and Prevention [CDC] Stage A), and 50 (34.0%) had at least one comorbidity. Upon release, 74 (50.3%) were on anti-retroviral therapy (ART). Prior to release, 124 (84.5%) of those receiving ART had a viral load of ≤200cp/ml. Following release, 12 (8.2%) cohort members died, with a crude incidence of 33.8/1000 person-years. All recorded deaths were males, with an incidence of 42.2/1000

person-years. Compared with the age-specific mortality rates for males in French Guiana, the standardized mortality ratio was 14.8. In multivariate analysis, factors associated with death were age and CD4 cell count (i.e. the cells killed by HIV) prior to release. Further information is available by contacting Dr. Florence Huber (florence.huber@ch-cayenne.fr).

15. Kariminia; Australia (5, 26-30)

This was a retrospective study using data linkage to examine the mortality risk and causes of death among a cohort of incarcerated adults. The cohort included all 85,203 individuals who had experienced full-time custody in New South Wales (NSW), Australia between 1 January 1988 and 31 December 2002. Death was identified by record linkage to the Australian National Death Index based on the following common identifiers: full name, sex, date of birth, and date of last contact with the prison system. The cohort was followed from the date of first imprisonment on or after 1 January 1988 until the date of death or the end date of the study (31 December 2002). Deaths from all major 'disease-related' and 'external' cause categories were analysed, and the standardised mortality ratios (SMR) were reported using age, sex, and calendar-specific death rates for the NSW population. Factors associated with death were also examined, as well as the risk of suicide and drug overdose soon after release from incarceration. The median age at the study entry for men was 27.2 years and for women 27.3 years. In total, there were 5,137 deaths (4,714 men, 423 women) during a median follow-up of 7.7 years. The median age at the time of death was 36.6 years for men and 32.7 years for women. All-cause SMR was 3.7 (95% CI: 3.6–3.8) in men and 7.8 (95% CI: 7.1–8.5) in women. Further information is available by contacting Dr. Azar Kariminia at the Kirby Institute, University of New South Wales (akariminia@kirby.unsw.edu.au).

16. Kinner (A); Australia (10, 31-37)

The Mortality After Release from Custody (MARC) study, led by Prof. Stuart Kinner, is a data linkage study of mortality in adults released from incarceration in the state of Queensland, Australia. It was funded by the NHMRC (APP456107). The cohort includes all persons released from full-time custody in Queensland from 1 January 1994 to 31 December 2007 (inclusive). Participant identities were linked probabilistically with Australia's National Death Index until 31 December 2007 and all known aliases were included in the linkage process. The cohort includes 42,015 individuals, although most analyses exclude those who died

during a subsequent episode of re-incarceration (n=45), leaving a valid sample size of 41,970. Of these, 2,158 (5.1%) died in the community during follow-up. The total follow-up time was 292,957 person-years (median 7.5 years per person), including community follow-up time (excluding subsequent episodes of imprisonment) of 270,394 person years (median 6.8 years per person). The sample is predominantly male (88%) and aged <40 at index release (80%; median age 31 years); Indigenous people are markedly over-represented in Australian prisons and constitute 19% of the cohort. Further information is available by contacting Prof. Stuart Kinner (s.kinner@unimelb.edu.au).

17. Kinner (B); Australia (38)

The Queensland Youth Justice Mortality study, led by Prof. Stuart Kinner, is a retrospective data linkage study in Queensland, Australia linking state youth justice records, state adult correctional records, and National Death Index records. Herein, we use only 13,188 individuals with adult correctional records between January 1994 and January 2017 who were aged 16 years or older on release from their first adult imprisonment. These individuals were followed up for a median of 7.9 years (IQR: 3.4 – 13.5 years; range: 0 – 23.0 years). During 114,930 person-years of follow-up, there was a total of 481 deaths. Further information is available by contacting Prof. Stuart Kinner (s.kinner@unimelb.edu.au).

18. Kouyoumdjian (A); Canada (39)

This study examined the causes and rates of mortality of all adults admitted to provincial correctional facilities in Ontario, Canada in 2000. Of 49,470 persons admitted to custody in that year, a total of 48,166 (97.4%) were linked with health administrative data, using deterministic linkage by provincial health card number or probabilistic linkage by name and date of birth. The linked cohort consisted of 43,419 men with 500,942 person-years of follow up, and 4,747 women with 57,260 person-years of follow up. A total of 34,595 person years (6.6% of the total for men) and 1,929 person-years (3.4% of the total for women) were spent in provincial custody during the follow-up period. During the follow-up period, 8.6% of the linked cohort (N=4,126) died either in custody or after release. Further information is available in Kouyoumdjian et al. (39) or by contacting Dr. Fiona Kouyoumdjian (kouyouf@mcmaster.ca).

19. Kouyoumdjian (B); Canada (40)

This study, led by Fiona Kouyoumdjian, involves data from all persons released from provincial correctional facilities in Ontario, Canada in 2010 who were successfully linked with provincial health administrative data (N= 48,861). Further information is available by contacting Dr. Fiona Kouyoumdjian (kouyouf@mcmaster.ca).

20. Lim; USA (41, 42)

In this study, the New York City Department of Health and Mental Hygiene evaluated the health of adults who were incarcerated in a New York City Department of Correction jail in 2001-05. Using matched administrative data, the retrospective cohort included 244,298 adults aged between 16 and 89 years. Of these, the mortality study focused on 200,493 adults released to the community during the study period (2001-05). Those with missing residence information, missing last jail release date, or those who died during incarceration without records in jail were excluded, leaving a final sample of 155,272 adults and 379,363 person-years in the community after release. Jail records were probabilistically matched to records from the death registry and single-adult homeless shelter registry, using first and last name, birth date, sex and Social Security Number. The cohort members, compared with the overall New York City population, were more likely to be younger, male and non-Hispanic black. During 2001-05, almost a half of all cohort members were incarcerated more than once and 10% stayed in a New York City single-adult homeless shelter. A total of 1,149 died after release, including 219 drug-related deaths, 219 homicides and 35 suicide deaths. Further information can be found in Lim et al. (41) or by contacting Dr. Sungwoo Lim (slim1@health.nyc.gov).

21. Morenoff; USA

The Michigan State Parole Cohort study, led by Dr. Jeffrey Morenoff, is based on detailed administrative records from the Michigan Department of Corrections (MDOC) on a cohort of 11,064 adults who were paroled from Michigan state prisons to the community during 2003. The cohort consists of people returning from state prisons (i.e., correctional institutions run by a state or the federal government that hold individuals serving felony

sentences, typically for two or more years, or those serving time for violation of post-prison community supervision, such as parole), but does not include those returning from county jails (i.e., institutions run by local cities and counties that hold people whose sentences are less than two years, or who are awaiting trial). As more than 90% of people released from Michigan's state prisons are put on parole, the cohort is very close to representative of people exiting prison. Data sources include MDOC records, Michigan State Police arrest records, Unemployment Insurance system records, and death records from the National Death Index, matched using names, social security numbers, and birthdates. The cohort is 92% male, 53% African American, 45% white, and 2% other (mostly Mexican American). At the time of their release, 18% were aged under 25 years. This study is funded by the National Institute on Child Health and Development of the National Institutes of Health and the National Science Foundation. Further information is available by contacting Dr. Jeffrey Morenoff (morenoff@umich.edu).

22. Pizzicato & Viner; USA

The Philadelphia Department of Prisons release study is a retrospective cohort study of 82,780 adults released to the community between 1 January 2010 and 30 December 2016. The Philadelphia Department of Prisons is a municipal jail system housing adults awaiting trial, sentencing, or transfer to state prisons, those serving shorter sentences (<2 years) and those who violated parole or probation. The study cohort included 82,780 individuals released to the community with 168,155 releases and 325,393 person-years of follow-up time. The median length of follow-up was 4.2 years. Individuals were linked to Philadelphia Medical Examiner's Office data to record overdose deaths and the Pennsylvania Department of Health, Vitals Record database to assess all-cause mortality. Of the individuals released, 2,522 (3%) died from any cause, of which 837 (33%) died from an overdose. This study was supported in part by the Applied Epidemiology Fellowship Program administered by the Council of State and Territorial Epidemiologists (CSTE) and funded through the Centers for Disease Control and Prevention (CDC) Cooperative Agreement Number 1U38OT000143-04 by the Substance Abuse and Mental Health Services Administration. Further information is available by contacting Lia Pizzicato (lia.pizzicato@phila.gov).

23. Preen (A); Australia (43)

This study involved population-based, retrospective data linkage between routinely-collected, administrative Department of Justice and Department of Health records in Western Australia (WA). Funded by the Australian Criminal Research Council, the overarching aim of the study was to describe the health problems experienced by adults after release from incarceration, in order to provide information to guide the provision of preventative and clinical services. The cohort consisted of 16,162 adults who were released from incarceration in WA between 1 January 1994 and 31 December 2001, inclusive. Linked data were extracted from the WA Department of Justice (for all custodial sentences), Hospital Morbidity Data System, Mental Health Information System and Mortality Register. Cohort members were followed for a minimum period of two years (to the end of 2003) with a total follow-up time of 69,714 person-years, of which 62,662 (89.9%) were spent in the community. The age of the cohort at the date of first release ranged from 16-87 years, with a mean age of 30 years. The composition of the cohort was 5.4% Aboriginal females, 29.8% Aboriginal males, 5.4% non-Aboriginal females and 58.5% non-Aboriginal males. A total of 698 deaths were recorded by the end of the study follow-up period. Further information is available by contacting Prof. David Preen (david.preen@uwa.edu.au).

24. Preen (B); Australia (44, 45)

This project comprised a population-based, retrospective cohort study involving data linkage between routinely-collected, administrative correctional and health services records in Western Australia (WA). The cohort consists of all adults convicted of one or more offences in WA aged 18–44 years (n=6,041) who commenced their first custodial sentence between 1 January 1985 and 31 December 1994 in one of WA's 13 prisons. A total of 645 deaths were recorded. Further information is available by contacting Prof. David Preen (david.preen@uwa.edu.au).

25. Ranapurwala; USA (46)

This is an ongoing retrospective cohort study of 229,274 formerly incarcerated adults released from prisons in North Carolina, US, between 1 January 2000 and 31 December 2015. Correctional data have been linked to North Carolina death records from 2000 to 2016 so that

each cohort member had a chance to accrue at least one year of post-release person-time. There were 387,913 prison releases during the study period, contributing 1,975,274 person-years of follow-up time. The median age at release was 34 years (range=18-92 years), 86% were males, most (60%) were non-White and half (50%) had no history of prior incarcerations. A total of 14,086 post-release deaths was recorded. The primary aim of this study is to examine how the opioid epidemic in the US affects formerly incarcerated adults, in relation to rates of opioid overdose deaths and predictors of opioid mortality. Further information is available by contacting A/Prof. Shabbar I. Ranapurwala (sirana@email.unc.edu).

26. Rosen; USA (47, 48)

This study linked North Carolina prison records from 1 January 1980 to 30 December 2004) with state death records from 1 January 1980 to 31 December 2005 to estimate the number of all-cause and cause-specific deaths among Black and White males aged 20 to 69 years following release from incarceration. Records were matched deterministically using the following four identifiers: surname, first name, date of birth, and the last four digits of the social security number. Records were also linked if they matched deterministically on three of the four identifiers, but differed by phonetically similar names or nicknames, or in instances when two of the three date of birth variables (e.g. month, day, year) and all remaining identifiers matched; these records accounted for about 12% of all linked records. The study cohort consisted of 168,001 male adults released from incarceration by the North Carolina Department of Correction Division of Prisons between 1980 and 2004. The median follow-up time from the most recent prison release until either death or the end of the observation period was 8.2 years (IQR=4.8–12.9 years), resulting in a total of 1,010,451 person-years at risk. The cohort was 52% black, the median incarceration time was 8.8 months (IQR: 3.4 – 26.6 months), and the median age of release was 31 years (IQR: 25 – 39 years). A total of 9,001 (8.0%) of cohort members died between 1980 and 1999. Further information is available by contacting A/Prof. David Rosen (david_rosen@med.unc.edu).

27. Slaunwhite & Zhao; Canada (49, 50)

The British Columbia Provincial Overdose Cohort is a collection of linked administrative data on overdose events that are combined with data about prescription medications, social

assistance programs, mental health service utilization, provincial incarceration history, and overall healthcare use. Included in the cohort is a 20% random reference sample of the population of British Columbia aged ≥18 years (N=834,067). Among this reference sample, 9,993 (1.2%) adults had a history of incarceration and are included in the MARIC study. Compared to those without a history of incarceration, the majority of adults with a history of incarceration are male (87% vs. 49%) and also have a younger mean age (37.2 vs 39.9, SD = 11.7 vs. 18.5). Further information is available by contacting Dr. Amanda Slaunwhite (amanda.slaunwhite@bccdc.ca) or by visiting the British Columbia Centre for Disease Control website (www.bccdc.ca/our-research/projects/overdose-cohort-data).

28. Somers; Canada (51)

Somers, Canada: This project, led by Prof. Julian Somers, consisted of an ongoing population-based provincial cohort of incarcerated adults (N=250,884 persons, 1997 to 2015) in British Columbia (BC), Canada. Information was obtained through data linkage between routinely collected, administrative correctional, social assistances and health services records in British Columbia (BC). The current study was restricted to participants who were released from provincial custody between January 1, 2007 and March 31, 2015. The eligible sample included 42,265 adults, which contributed a total of 125,312 custody episodes (on average 3 episodes per participant). The vast majority of this custody cohort was male (37,176; 87.2%). Moreover, 24,945 (58.5%) were Caucasian, 9,480 (22.2%) were Aboriginals and the rest belong to other ethnicities. The average age of this cohort was 35.2 years (SD: 11. 3 yrs.) at the time of initial release from custody. A total of 1,430 participants died during the study period (January 1, 2007 and March 31, 2015), 256 of which occurred in the first 12 weeks (84 days) of post-release. Further information is available by contacting Prof. Julian Somers (jsomers@sfu.ca).

29. Spaulding; USA (52, 53)

The cohort in this study consisted of 23,510 adults incarcerated in any prison facility run by the Department of Corrections in Georgia, USA, on 30 June 1991, of whom 20,743 (88.2%) were released on at least one occasion and were included in the cohort for the MARIC study. Cohort members were predominantly male (94%) and black (67%). The mean age of

the cohort on 30 June 1991 was 24 years (SD: 9 years). Survival was examined by linking cohort members retrospectively with the National Death Index to provide data on all deaths through 31 December 2006 and again through 31 December 2010. Mortality data included the date of death and the ICD-9/ICD-10 codes for the primary and secondary causes of death. A total of 3582 cohort members (16%) died between 1991 and 2010. Most (66%) had one release during the follow-up period, and one in ten remained continuously incarcerated. Further information is available by contacting Dr. Anne Spaulding (aspauld@emory.edu).

References to the supplmentary material

1. Riley RD, Lambert PC, Abo-Zaid G. Meta-analysis of individual participant data: rationale, conduct, and reporting. Bmj. 2010;340:c221.

2. Bazazi AR, Wickersham JA, Wegman MP, Culbert GJ, Pillai V, Shrestha R, et al. Design and implementation of a factorial randomized controlled trial of methadone maintenance therapy and an evidence-based behavioral intervention for incarcerated people living with HIV and opioid dependence in Malaysia. Contemporary Clinical Trials. 2017;59:1-12.

3. Binswanger IA, Blatchford PJ, Mueller SR, Stern MF. Mortality after prison release: opioid overdose and other causes of death, risk factors, and time trends from 1999 to 2009. Ann Intern Med. 2013;159(9):592-600.

4. Binswanger IA, Stern MF, Deyo RA, Heagerty PJ, Cheadle A, Elmore JG, et al. Release from prison - a high risk of death for former inmates. New England Journal of Medicine. 2007;356(2):157-65.

5. Merrall EL, Kariminia A, Binswanger IA, Hobbs MS, Farrell M, Marsden J, et al. Meta-analysis of drug-related deaths soon after release from prison. Addiction. 2010;105(9):1545-54.

6. Binswanger IA, Blatchford PJ, Lindsay RG, Stern MF. Risk factors for all-cause, overdose and early deaths after release from prison in Washington state. Drug and alcohol dependence. 2011;117(1):1-6.

7. Wortzel HS, Blatchford P, Conner L, Adler LE, Binswanger IA. Risk of death for veterans on release from prison. Journal of the American Academy of Psychiatry and the Law. 2012;40(3):348.

8. Calcaterra S, Blatchford P, Friedmann PD, Binswanger IA. Psychostimulant-related deaths among former inmates. Journal of addiction medicine. 2012;6(2).

9. Binswanger IA, Stern MF, Yamashita TE, Mueller SR, Baggett TP, Blatchford PJ. Clinical risk factors for death after release from prison in Washington State: a nested case–control study. Addiction. 2016;111(3):499-510.

10. Binswanger IA, Blatchford PJ, Forsyth SJ, Stern MF, Kinner SA. Epidemiology of Infectious Disease–Related Death After Release from Prison, Washington State, United States, and Queensland, Australia: A Cohort Study. Public Health Reports. 2016;131(4):574-82.

11. Bukten A, Stavseth MR, Skurtveit S, Tverdal A, Strang J, Clausen T. High risk of overdose death following release from prison: variations in mortality during a 15-year observation period. Addiction. 2017; DOI: 10.1111/add.13803.

12. Chen CY, Wu PN, Su LW, Chou YJ, Lin KM. Three-year mortality and predictors after release: a longitudinal study of the first-time drug offenders in Taiwan. Addiction. 2010;105(5):920-7.

13. Culbert JG, Crawford FW, Murni A, Waluyo A, Bazazi AR, Sahar J, et al. Predictors of mortality within prison and after release among persons living with HIV in Indonesia. Research and Reports in Tropical Medicine. 2017;8:25-35.

14. Degenhardt L, Larney S, Kimber J, Gisev N, Farrell M, Dobbins T, et al. The impact of opioid substitution therapy on mortality post-release from prison: retrospective data linkage study. Addiction. 2014;109(8):1306-17.

15. Larney S, Gisev N, Farrell M, Dobbins T, Burns L, Gibson A, et al. Opioid substitution therapy as a strategy to reduce deaths in prison: retrospective cohort study. BMJ open. 2014;4(4):e004666.

16. Kimber J, Larney S, Hickman M, Randall D, Degenhardt L. Mortality risk of opioid substitution therapy with methadone versus buprenorphine: a retrospective cohort study. The Lancet Psychiatry. 2015;2(10):901-8.

17. Gisev N, Shanahan M, Weatherburn DJ, Mattick RP, Larney S, Burns L, et al. A cost-effectiveness analysis of opioid substitution therapy upon prison release in reducing mortality among people with a history of opioid dependence. Addiction. 2015;110(12):1975-84.

18. Gisev N, Larney S, Kimber J, Burns L, Weatherburn D, Gibson A, et al. Determining the impact of opioid substitution therapy upon mortality and recidivism among prisoners: A 22 year data linkage study. Trends and Issues in Crime and Criminal Justice. 2015(498):1.

19. Dirkzwager A, Nieuwbeerta P, Beijersbergen K, Bosma A, de Cuyper R, Doekhie J, et al. Cohort profile: the prison project—a study of criminal behavior and life circumstances before, during, and after imprisonment in the Netherlands. Journal of Developmental and Life-Course Criminology. 2018;4(1):120-35.

20. Dolan KA, Shearer J, White B, Zhou J, Kaldor J, Wodak AD. Four-year follow-up of imprisoned male heroin users and methadone treatment: mortality, re-incarceration and hepatitis C infection. Addiction. 2005;100(6):820-8.

21. Chang Z, Lichtenstein P, Larsson H, Fazel S. Substance use disorders, psychiatric disorders, and mortality after release from prison: a nationwide longitudinal cohort study. The Lancet Psychiatry. 2015;2(5):422-30.

22. Graham L, Fischbacher CM, Stockton D, Fraser A, Fleming M, Greig K. Understanding extreme mortality among prisoners: a national cohort study in Scotland using data linkage. The European Journal of Public Health. 2015;25(5):879-85.

23. Bird SM, Fischbacher CM, Graham L, Fraser A. Impact of opioid substitution therapy for Scotland's prisoners on drugs-related deaths soon after prisoner-release. Addiction. 2015.

24. Huang YF, Kuo HS, Lew-Ting CY, Tian F, Yang CH, Tsai TI, et al. Mortality among a cohort of drug users after their release from prison: an evaluation of the effectiveness of a harm reduction program in Taiwan. Addiction. 2011;106(8):1437-45.

25. Huber F, Merceron A, Madec Y, Gadio G, Pastre A, Coupez I, et al. High mortality among male HIV-infected patients after prison release: ART is not enough after incarceration with HIV. PloS one. 2017;12(4):e0175740.

26. Kariminia A, Law MG, Butler TG, Corben SP, Levy MH, Kaldor JM, et al. Factors associated with mortality in a cohort of Australian prisoners. Eur J Epidemiol. 2007;22(7):417-28.

27. Kariminia A, Law MG, Butler TG, Levy MH, Corben SP, Kaldor JM, et al. Suicide risk among recently released prisoners in New South Wales, Australia. Medical journal of Australia. 2007;187(7):387.

28. Kariminia A, Butler TG, Corben SP, Levy MH, Grant L, Kaldor JM, et al. Extreme cause-specific mortality in a cohort of adult prisoners—1988 to 2002: a data-linkage study. International journal of Epidemiology. 2007;36(2):310-6.

29. Kariminia A, Butler T, Corben S, Kaldor J, Levy M, Law M. Mortality among prisoners: how accurate is the Australian National Death Index? Australian and New Zealand journal of public health. 2005;29(6):572-5.

30. Kariminia A, Butler T, Jones J, Law M. Causes of death among Indigenous persons during and after release from prisons in New South Wales, Australia. Aust NZ J Publ Heal 2012;36:274-280. .

31. Kinner SA, Forsyth SJ. Development and Validation of a National System for Routine Monitoring of Mortality in People Recently Released from Prison. PloS one. 2016;11(6):e0157328.

32. Spittal MJ, Forsyth S, Pirkis J, Alati R, Kinner SA. Suicide in adults released from prison in Queensland, Australia: a cohort study. Journal of epidemiology and community health. 2014;68(10):993-8.

33. Forsyth SJ, Alati R, Ober C, Williams GM, Kinner SA. Striking subgroup differences in substance-related mortality after release from prison. Addiction. 2014;109(10):1676-83.

34. van Dooren K, Kinner SA, Forsyth S. Risk of death for young ex-prisoners in the year following release from adult prison. Australian and New Zealand journal of public health. 2013;37(4):377-82.

35. Kinner SA, Forsyth S, Williams G. Systematic review of record linkage studies of mortality in ex-prisoners: why (good) methods matter. Addiction. 2013;108(1):38-49.

36. Andrews JY, Forsyth S, Wade J, Kinner SA. Sensitivity of a national coronial database for monitoring unnatural deaths among ex-prisoners in Australia. BMC research notes. 2011;4(1):450.

37. Kinner SA, Preen DB, Kariminia A, Butler T, Andrews JY, Stoové M, et al. Counting the cost: estimating the number of deaths among recently released prisoners in Australia. Medical Journal of Australia. 2011;195(2):64-8.

38. Tibble H, Di Law H, Spittal MJ, Karmel R, Borschmann R, Hail-Jares K, et al. The importance of including aliases in data linkage with vulnerable populations. BMC medical research methodology. 2018;18(1):76.

39. Kouyoumdjian F, Kiefer L, Wobeser W, Hwang S. Mortality over 12 years of follow up in people admitted to provincial custody in Ontario: A retrospective cohort study. Canadian Medical Association Journal Open. 2016;4(2):E153-E61.

40. Kouyoumdjian FG, Cheng SY, Fung K, Orkin AM, McIsaac KE, Kendall C, et al. The health care utilization of people in prison and after prison release: A population-based cohort study in Ontario, Canada. PloS one. 2018;13(8):e0201592.

41. Lim S, Seligson AL, Parvez FM, Luther CW, Mavinkurve MP, Binswanger IA, et al. Risks of drug-related death, suicide, and homicide during the immediate post-release period among people released from New York City jails, 2001-2005. Am J Epidemiol. 2012;175(6):519-26.

42. Lim S, Harris TG, Nash D, Lennon MC, Thorpe LE. All-cause, drug-related, and HIV-related mortality risk by trajectories of jail incarceration and homelessness among adults in New York City. American journal of epidemiology. 2015:kwu313.

43. Sodhi-Berry N, Knuiman M, Alan J, Morgan VA, Preen DB. Pre-sentence mental health service use predicts post-sentence mortality in a population cohort of first-time adult offenders. Soc Psychiatry Psychiatr Epidemiol. 2015;50(1):109-24.

44. Stewart L, Henderson C, Hobbs M, Ridout S, Knuiman M. Risk of death in prisoners after release from jail. Australian and New Zealand journal of public health. 2004;28(1):32-6.

45. Jama-Alol KA, Malacova E, Ferrante A, Alan J, Stewart L, Preen D. Influence of offence type and prior imprisonment on risk of death following release from prison: a whole-population linked data study. International journal of prisoner health. 2015;11(2):108-18.

46. Ranapurwala SI, Shanahan ME, Alexandridis AA, Proescholdbell SK, Naumann RB, Edwards Jr D, et al. Opioid Overdose Mortality Among Former North Carolina Inmates: 2000–2015. American journal of public health. 2018;108(9):1207-13.

47. Rosen DL, Schoenbach VJ, Wohl DA. All-cause and cause-specific mortality among men released from state prison, 1980-2005. Am J Public Health. 2008;98(12):2278-84.

48. Rosen DL, Wohl DA, Schoenbach VJ. All-cause and cause-specific mortality among black and white North Carolina state prisoners, 1995–2005. Annals of epidemiology. 2011;21(10):719-26.

49. MacDougall L, Smolina K, Otterstatter M, Ko M, Godfrey D, Zhao B, et al. Development and characteristics of the provincial overdose cohort in British Columbia, Canada. International Journal of Population Data Science. 2018;3(4).

50. Otterstatter MC, Crabtree A, Dobrer S, Kinniburgh B, Klar S, Leamon A, et al. Patterns of health care utilization among people who overdosed from illegal drugs: a descriptive analysis using the BC Provincial Overdose Cohort. Health promotion and chronic disease prevention in Canada: research, policy and practice. 2018;38(9):328-33.

51. McCandless LC, Stewart LC, Rempel ES, Venners SA, Somers JM. Criminal justice system contact and mortality among offenders with mental illness in British Columbia: an assessment of mediation. Journal of epidemiology and community health. 2014:jech-2013-203705.

52. Spaulding AC, Seals RM, McCallum VA, Perez SD, Brzozowski AK, Steenland NK. Prisoner survival inside and outside of the institution: implications for health-care planning. Am J Epidemiol. 2011;173(5):479-87.

53. Spaulding AC, Sharma A, Messina LC, Zlotorzynska M, Miller L, Binswanger IA. A comparison of liver disease mortality with HIV and overdose mortality among Georgia prisoners and releasees: a 2-decade cohort study of prisoners incarcerated in 1991. American journal of public health. 2015;105(5):e51-e7.
